# Interleukin-10 and Transforming Growth Factor-β in Early and Late Lesions of Patients with *Leishmania major* induced Cutaneous Leishmaniasis

**Published:** 2012

**Authors:** SH Hejazi, SG Hoseini, SH Javanmard, SH Zarkesh, A Khamesipour

**Affiliations:** 1 Skin Diseases and Leishmaniasis Research Center, Department of Parasitology & Mycology, School of Medicine, Isfahan University of Medical Sciences, Isfahan, Iran; 2 Department of Parasitology & Mycology, School of Medicine, Isfahan University of Medical Sciences, Isfahan, Iran; 3 Physiology Research Center, Department of Physiology, Isfahan University of Medical Sciences, Isfahan, Iran; 4 Department of Immunology, School of Medicine, Isfahan University of Medical Sciences, Isfahan, Iran; 5 Center for Research in Skin Disease and Leprosy, Tehran University of Medical Sciences, Tehran, Iran

**Keywords:** Cutaneous leishmaniasis, *Leishmania major*, Interleukin-10, Transforming growth factor-β, Immunofluorescence

## Abstract

**Background:**

Cutaneous leishmaniasis is a neglected parasitic disease, which imposes massive human distress and financial costs to the endemic countries. Better understanding of host immune response to the parasite leads to helpful strategies for disease control. Interleukin (IL)-10 and transforming growth factor (TGF)-β are important immune regulatory cytokines, which appear to develop non-healing forms of leishmaniasis. However, there is little information about the function of IL-10 and TGF-β in old world cutaneous leismaniasis. The aim of this study was to analyze the role of IL-10 and TGF-β in human cutaneous leishmaniasis due to *Leishmania major* infection.

**Methods:**

Biopsies were obtained from lesions of twenty proven cases of *L. major* induced cutaneous leishmaniasis. IL-10 and TGF-β positive cells were detected by immunofluorescence staining of frozen sections and compared between two groups of patients with early and late lesions.

**Results:**

The mean percentage of IL-10 positive cells were significantly (*P*= 0.035) higher in late lesions (0.51±0.24) than early ones (0.15±0.07). Similar results were obtained for TGF-β with mean percentages of 0.16±0.05 and 0.53±0.28 in early and late lesions respectively (*P*= 0.008).

**Conclusion:**

IL-10 and TGF-β are present in lesions of *L. major* induced cutaneous leishmaniasis and contribute to the pathogenesis of long lasting disease forms.

## Introduction

Cutaneous leishmaniasis (CL) is a common destructive skin infection caused by different species of *Leishmania* parasite. Endemic foci for CL show distinct clinical pictures of disease due to particular parasite species and/or population features (1). The old world CL occurs in central and west Asia, India, and Africa, in contrast to the new world CL that occurs mainly in western countries. Old world CL is one of the most neglected diseases of the world owing to the paucity of investigations for disease prevention and treatment (2). The causative agents of old world CL are *L. major* and *L. tropica*, which induce wet, early ulcerative and dry, late ulcerative forms of disease respectively. *L. major* infection is the zoonotic form of the disease (ZCL) (1).

Since the anti *Leishmania* chemotherapy does not meet an effective, low side effect treatment protocol especially for long lasting and refractory cases, early quests for alternative treatments have begun (3). Clear understanding of disease pathophysiology may help to achieve this aim and the most important aspect of this is perhaps the host immune response to the parasite.

What we know about the immune-pathogenesis of old world CL generally comes from animal models of infection. The T cell response to infection appears to determine the outcome of infection toward healing or nonhealing forms of disease with T helper (h)1 response causing protection and Th2 response inducing exacerbation (4). It is now obvious that besides Th1 /Th2 profile of immune response, the immune regulatory factors including regulatory T cells and regulatory cytokines, interleukin (IL)-10 and transforming growth factor (TGF)-β, play an important role in development and chronicity of CL lesions at least in animal models of *Leishmania* infection (5, 6).

IL-10 and TGF-β are two potent immunosuppressive cytokines, which act via distinct pathways to modulate excessive immune responses and immunopathology in allergy, autoimmunity, and infectious disease (7, 8). IL-10 and TGF-β have inhibitory effects on macrophages which are the main targets of *Leishmania* parasite, reduce their ability to kill the parasite and their antigen presentation to effector T cells. Moreover, they regulate effector T cells directly via inhibiting their proliferation and cytokine production (7, 9). Secretion of IL-10 and TGF-β are also important mechanisms of regulatory T cell mediated immune suppression (10).

Several studies on new world CL have revealed that IL-10 and TGF-β are associated with chronic forms of the disease (11) or long lasting atypical lesions (12).

There are few studies which have addressed the contribution of IL-10 to the pathophysiology of old world CL and these studies showed no relevance between their result, moreover no report of TGF-β measurement in old world CL is available currently.

In order to reappraise the role of IL-10 and TGF-β in chronicity of old world ZCL, their expression was assessed in lesions of ZCL patients by means of immunofluorescence (IF) staining of frozen sections and the frequency of positive cells for these cytokines were compared between two groups of patients with early and late ZCL lesions.

## Materials and Methods

### Patients

Twenty patients with active CL lesions were selected from those referred to Centre for Research in Skin Diseases and Leishmaniasis, Iran, Isfahan University of Medical Sciences. Informed consents were completed by all the subjects and the study was approved by ethical committee of Isfahan University of Medical Sciences, Ministry of Health, Iran. Selected cases were divided in two groups of patients with early and late lesions based on duration of disease prior to the time of taking biopsies. Early lesions (n= 10) were those with duration of less than four months and late lesions (n= 10) were those which appeared at least six months before the study. Parasitological diagnosis was based on direct microscopy and the patients with a history of chronic internal or cutaneous disease or evidence of bacterial infection of the lesions were excluded from the study. The causative agents of lesions were identified as *L. major* by means of high-resolution melting analysis of 7SL RNA gene of *Leishmania* parasite in biopsy specimens as described elsewhere (13). Some characteristics of patients are summarized in Table 1.

**Table 1:** Clinical characterization of the patients

	Patients with early lesions	Patients with late lesions
**Age**		
year, mean (range)	23.1 (16-29)	34.7 (16-58)
**Sex**		
male/female	10/0	9/1
**Duration of lesions**		
month, mean(range)	2.3(1.5-3)	8.7(6-14)
**Lesion localization**		
upper limb	7	7
lower limb	2	3
trunk	1	0
**Therapy**	0	4

### Biopsy collection

3.5 mm punch biopsies were taken from locally anaesthetized border of lesions and skin defects were closed with stitches. Samples were fixed in 4% paraformaldehyde overnight and dehydrated in 30% sucrose solution for 24 hours at 4°C. Fixed biopsies were embedded in OCT compound (Tissue-Tek, 27050) and six-micrometer cryostat sections were cut using a Leica 1800 cryocut (Leica, Germany). Sections were placed on poly L-lysine (Sigma, P8920) coated slides and stored at -80 until used. Tonsils were obtained from tonsillectomy surgeries and processed as patient biopsies.

### Immunofluorescent staining

The following antibodies were used for IF staining of prepared sections: Anti IL-10 mouse monoclonal antibody (Santa Cruz Biotechnology, sc-8438, dilution: 1/30), Anti TGF-β mouse monoclonal antibody (Santa Cruz Biotechnology, sc-52830, dilution: 1/30) and TRITC-conjugated goat anti-mouse IgG (Sigma, T7782, dilution 1:50).

After rehydration with PBS nonspecific sites were blocked with 10mg/ml bovine serum albumin (BSA). Permeabilization was done using 0.4% Triton X-100 for 45 min. Then sections were incubated with anti TGF-β or anti IL-10 antibodies for 1 h at 37°C followed by overnight incubation at room temperature. TRITC-conjugated secondary antibody was applied for 45 min at 37°C then sections were counter stained with 4,6-diamidino-2-phenylindole (DAPI) (Sigma, D9542). Negative control was omission of primary antibodies and tonsillar sections served as positive controls.

### Microscopy and Quantification

The sections were visualized using fluorescent microscopy system (Olympus, Bx-51, Japan) and photographed with a digital camera (Olympus, DP-72, Japan) using Cell*A software (Olympus Soft Imaging Solution GmbH, Germany). Sections were coded and analyzed blindly. Target cells were examined in ten representative fields of at least two distinct stained sections under X400 magnification, which corresponded to an area of one mm^2^. Each field was photographed two times; one for specific labeled antibody and another for DAPI by switching filter and light intensity. “Manual tag” option of the software was used to count stained cells over the images.

The results were normalized to the total number of stained nuclei in each sample. For each sample, three representative fields of DAPI staining were selected and cell nuclei were counted over them and the average of cell number in one mm^2^ was calculated. The data was demonstrated as the percentage of IL-10^+^ or TGF-β^+^ cells relative to total cell counts.

### Statistical analysis

Statistical analyzes were carried out using SPSS 16.0 software. To compare the frequency of positive cells between groups Mann-Whitney U test was used. All tests were 2-tailed and *P* values of less than 0.05 were considered significant. Results are demonstrated as mean ± standard deviation.

## Results

### Frequency of IL-10 positive cells

IL-10^+^ cells were found in all biopsies but great variation among samples was seen in number of positive cells. Mean percentage of IL-10^+^ cells was 0.15±0.07 and 0.51±0.24 in early and late lesions respectively, which showed a meaningful higher percentage in late lesions than early ones with a *P* value of 0.035.

**Fig. 1:**
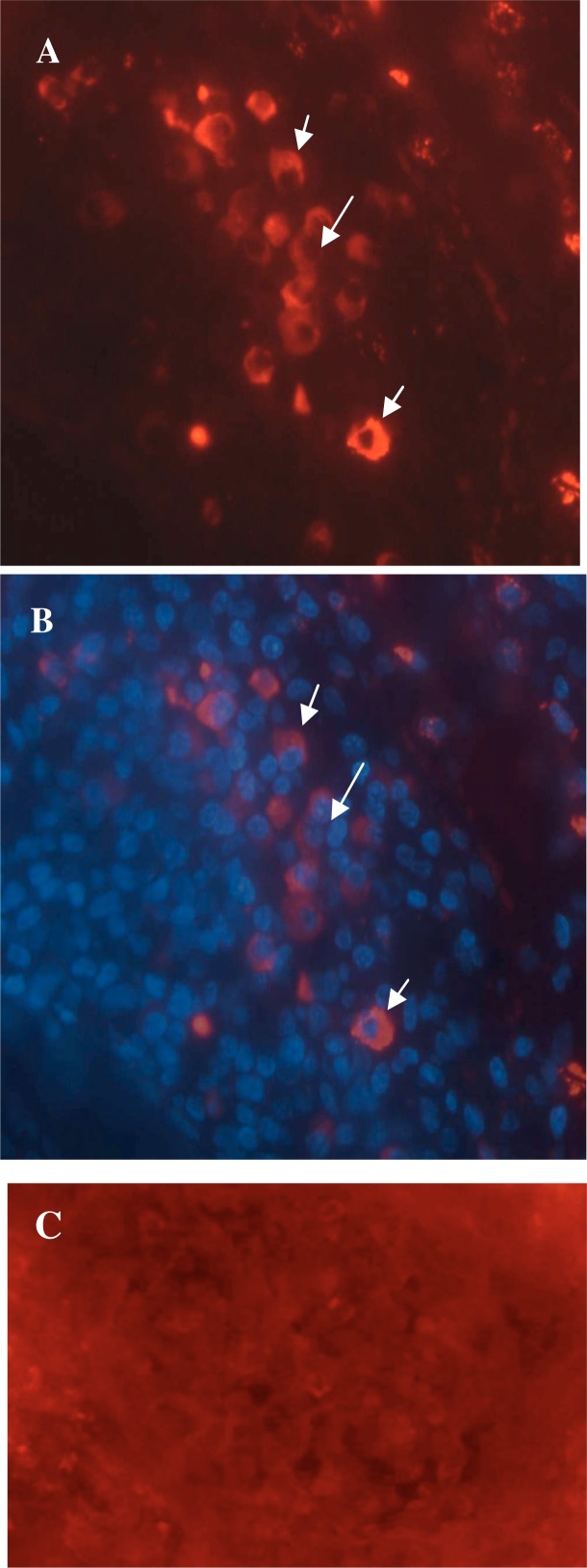
Immunofluorescence staining of IL-10 in a representative sample. A: IL-10 positive cells which show red cytoplasmic staining. B: DAPI staining of cellular nuclei. C: negative control for IL-10 and TGF-β staining which is processed similar to target sections except for omitting primary antibody. Original magnification is X400

IL-10^+^ cells were mainly seen in mid dermis and rarely in epidermis. In tonsillar sections, many IL-10^+^ cells were found and mostly were placed intra-follicular (Fig. 1).

### Frequency of TGF-β positive cells

**Fig. 2:**
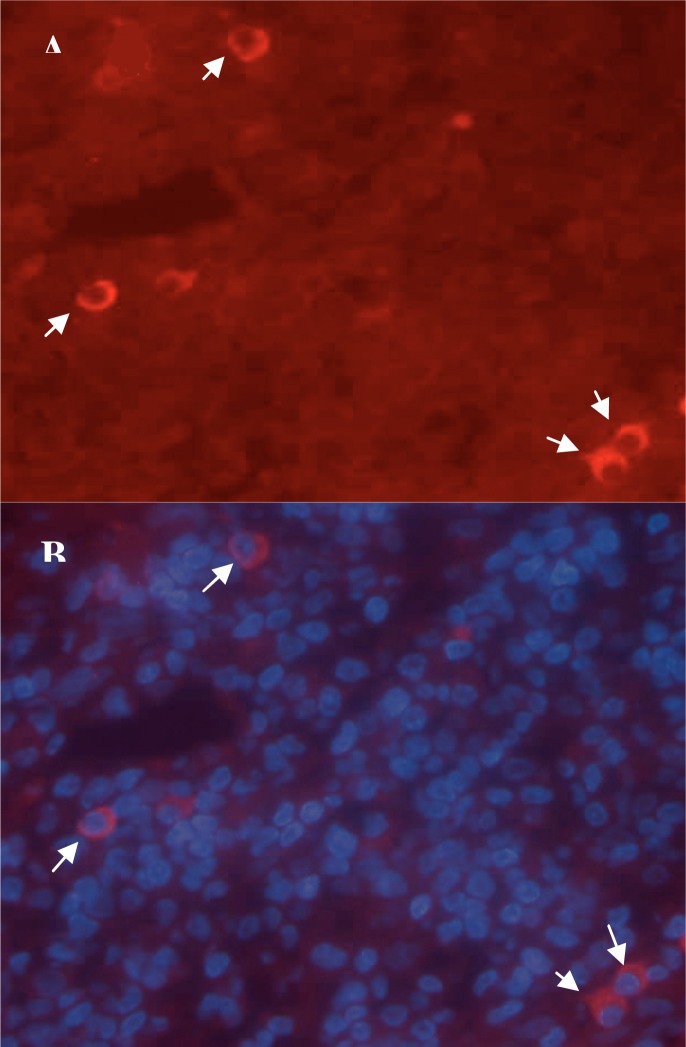
Immunofluorescence staining of TGF-β in a representative sample. A: TGF-β positive cells which show red cytoplasmic staining. B: DAPI staining of cellular nuclei. Original magnification is X400

All sections showed positive staining for TGF-β. Late lesions had significantly (*P*= 0.008) upper percentage of positive cells (0.53±0.28) than early ones (0.16±0.05). Like IL-10, epidermis was rarely stained positive for TGF-β. In tonsillar sections, TGF-β^+^ cells were placed extra-follicular (Fig. 2).

## Discussion

In this study, IL-10 and TGF-β were identified by IF method in biopsy samples of ZCL patients. It was shown that the cells positive for TGF-β and IL-10 were extensively higher in late lesions of old world CL than early ones which indicates that they play a considerable role in chronicity of the disease.

Several studies demonstrated the importance of IL-10 in exacerbation of CL in mouse models. It was shown that BALB/c mice treated with anti-IL-10R antibody controlled infection better (14). Moreover resistant mice expressing an IL-10 transgene (15) or treated with a recombinant adenovirus expressing IL-10 (16) produced a non-healing phenotype when infected with *L. major*. IL-10 was found to play an essential role in parasite persistence in genetically resistant C57BL/6 mice after spontaneous healing of their dermal lesions (17).

In a study of local immune response to *L. major* infection Louzir et al. showed that unfavorable evolution of the lesions was positively associated with high intralesional IL-10 (18). In addition, in localized CL due to *L. mexicana* a significant increase was observed in the expression of IL-10 and TGF-β in lesions with duration of more than four months (11). In American CL number of IL-10 and TGF-β secreting cells were more in long lasting lesions compared with acute lesions (12). These reports are in agreement with our results, although the degree of contribution of IL-10 and TGF-β to the pathogenesis of chronic disease appears to be different in various CL types. For example in *L. guyanensis* infection high intralesional IL-10 was predictive of poor prognosis (19) but level of IL-10 mRNA expression was not significantly different in acute and chronic cases (20).

Louzir et al. found IL-10 mRNA in only 70% of biopsies by means of quantitative PCR (18) but Gaafar et al. used imm-unohistochemistry (IHC) to localize IL-10 secreting cells and could detect the cytokine in all sections studied (21). All experiments which used immunohistological methods (12, 21, 22) could localize IL-10 secreting cells in different CL type lesions and their results are comparable with results of this study. The IF method used in this study allowed easier quantification of stained cells than IHC method.

Our results are however somewhat different with several other studies on old world CL (23–25) which detected no IL-10 secretion from the stimulated PBMC of healing and nonhealing CL patients. The reason for this difference may be the usage of PBMC stimulation rather than in situ cytokine assays.

The inflammatory cells that infiltrate the CL lesions include T cells, B cells, macrophages, Langerhans’ cells (26), and epidermal keratinocytes (27), can produce IL-10. Recently more emphasize is on regulatory T cells and IL-10 secreting Th1 cells as the sources of IL-10 in pathophisiology of several chronic inflammatory and infectious diseases (28, 29).

The exact cellular sources of IL-10 could not be determined in this study; however numerous reports of high intralesional interferon (IFN)-γ in both acute and chronic CL (18, 21) are available and recently Th1 cells that co-produce IL-10 and IFN-γ has been identified as important suppressors of the immune response in a non heal model of CL (30). These reports propose that Th1 cells are at least one of the main sources of IL-10 in clinical CL besides IL-10 producing regulatory T cells, which were characterized in CL models previously (5). Since persistent parasite stimulation is crucial to generate IL-10 secreting Th1 cells (28) and to maintain parasite specific regulatory T cells (31), these possible sources of IL-10 are probably rare in circulation and among PBMC. This may explain the dissimilarity between in situ and systemic cytokine assays in different studies of old world CL.

The effect of TGF-β on *Leishmania* has been vigorously studied in vitro and in vivo and reviewed in previous papers (32). Mouse and human macrophages infected with different *Leishmania* species actively produced TGF-β. The amount of TGF-β produced by macrophages correlated with both the strain virulence and the multiplication of parasites within the macrophages so TGF-β secretion is considered as a virulence factor for *Leishmania* parasite (32). TGF-β is synthesized and secreted as a latent form that needs activation to be functional so the protein analysis experiments of TGF-β are more reliable than gene expression studies (33).

The presence of TGF-β in lesions of acute patients in this and other mentioned studies and the constant function of TGF-β in skin immunity and wound healing (34) implicate a role for TGF-β in the initiation and establishment of *Leishmania* infection. Indeed IL-10 and TGF-β provide a suppressive milieu in the site of *Leishmania* infection, which promotes parasite persistent and replication, as well as prevention of excessive immunopathology in different stages of infection (9). This environment also supports the generation of regulatory T cell subsets and further suppression of effector T cells (10).

The variation observed in the frequency of positive cells among samples was previously reported in nearly all in situ human studies (11, 12, 18, 21) and may be due to different contact of subjects with environmental stimuli, infections and /or variable stages of the disease.

To the best of our knowledge, it is the first study of the role of TGF-β in old world ZCL and a revaluation of local IL-10 importance in this disease. The results of this study showed that not only IL-10 and TGF-β are constant elements of ZCL immune profile but also they play a considerable role in chronicity of the disease. These findings have to be translated in to the therapeutic strategies for better control of ZCL mainly the resistant forms.
